# Outcomes of Cataract Surgeries Over 16 Years in Camps Held by Al Basar International Foundation in 38 Underdeveloped Countries

**DOI:** 10.4103/0974-9233.80701

**Published:** 2011

**Authors:** Adel A. Rushood

**Affiliations:** Department of Ophthalmology, College of Medicines, King Faisal University, Dammam, Saudi Arabia

**Keywords:** Al-Basar International Foundation, Cataract Backlog, Cataract Blindness, Eye Camp

## Abstract

**Purpose:**

To evaluate the outcomes of 16 years of eye campaigns in 38 countries in Africa, Asia and the Middle East

**Materials and Methods:**

A descriptive, retrospective study using the Al-Basar International Foundation (BIF) records. BIF is a non-governmental, non-religious philanthropic organization working in the field of the prevention of blindness since 1989. Having its base in Saudi Arabia and working mainly in Asia and Africa. Study variables included the causes of diminished vision, outcomes of eye surgeries, number of camps, patients assessed, surgeries performed, intraocular lenses (IOLs) implanted, spectacles distributed, general outcomes of campaigns, and other variables.

**Results:**

Between the periods of November 1989 and June 2006, BIF conducted 620 eye camps. These camps were conducted by ophthalmologists with expertise of working in eye camps with limited resources and harsh environmental conditions. Over two million people were examined and/or treated, and 186, 765 surgeries were performed. Nearly 100,000 IOLs were implanted and more than 140,000 spectacles were prescribed and distributed. The majority of these activities (74%) took place in Asia and the Middle East. The best corrected visual acuity achieved (BCVA) was ranked good (6/6 -6/18) in 59% of patients and borderline (BCVA 6/18 - 6/60) in 35% and poor (BCVA <6/60) in less than 6% of patients based on World Health Organization (WHO) criteria.

**Conclusion:**

Quality assured eye campaigns held by BIF helped the most needy countries and people. Intensive volunteer cataract programs and surgeries such as those provided by the BIF add significant support to the efforts of the WHO and International Agency for the Prevention of Blindness in fighting blindness.

## INTRODUCTION

Worldwide there are approximately 37 million blind individuals and more than 124 million who are visually disabled.^1^ It is estimated that an additional 75 million men, women and children will be blind by the year 2020 if appropriate measures are not taken to address this health burden.^2^ Seventy-five percent of blindness in the world is avoidable and treatable.^3^

The right to sight is a global initiative of the World Health Organization (WHO) and the International Agency for the Prevention of Blindness (IAPB).^4^ Publicized under the initiative “Vision 2020,”^2^ its objective is to eliminate the main causes of avoidable blindness by the year 2020. The combination of governmental and non-governmental agencies (NGOs) to facilitate the planning, development, and implementation of sustainable eye care programs will help to achieve this goal. Such primary health care programs are based on the three core strategies of disease control, human resource development and infrastructure development.

Over the next two decades, the successful implementation of Vision 2020 will, hopefully, prevent blindness in an estimated 100 million people. NGOs play a crucial role and are major stakeholders of the prevention of blindness. Blindness prevention projects in many developed and developing countries as well as official programs are delivered by various NGOs.

Al-Basar International Foundation (BIF), a charitable non-governmental non-religious organization, registered in many countries, has been working for the prevention and eradication of blindness and blinding diseases since 1990. It has a comprehensive “Blindness Control Program” which consists of operating hospitals and conducting mobile eye services (eye camps). Mobile eye services (MES) refers to quality assured eye camps where screening, treating, prescribing and dispensing eye spectacles, and surgical management of cataract and other eye conditions are performed.

The goals of BIF are the provision of quality eye care, local workforce support, logistic base for eye camps, and creating an awareness of blindness.

This paper reports and discusses the outcomes of 16 years of eye campaigns in 38 countries. This report fills an important gap in the world literature and consolidates the efforts of the “right to sight” and the implementation of Vision 2020.

## MATERIALS AND METHODS

This is a descriptive, retrospective study based on the records of BIF. The study area comprised 38 countries from Africa, Asia, and the Middle East.

The study populations were patients presenting with diminished vision (DV) who sought treatment in 620 eye camps in 38 countries. The patients were mostly from the low socio-economic classes who could not afford the normal avenues of treatment.

The total number of patients who sought treatment at the camps was computed.

Study variables included causes of DV, outcomes of eye surgeries, number of camps, patients assessed, surgeries performed, number of intraocular lenses (IOLs) implanted, glasses dispensed, general outcomes of campaigns, and other variables.

The camp usually takes 7–10 days. The first 3 days are for screening, managing the non-surgical cases as well as dispensing reading glasses. Patients requiring surgery are admitted to the in-patient wards. Ruling out any contraindications such as infection or advanced glaucoma, diagnoses confirmations is performed by senior BIF ophthalmologists. In the ward, systemic blood pressure and glucose levels in urine were checked to ensure overall systemic health.

**Figure 1:**
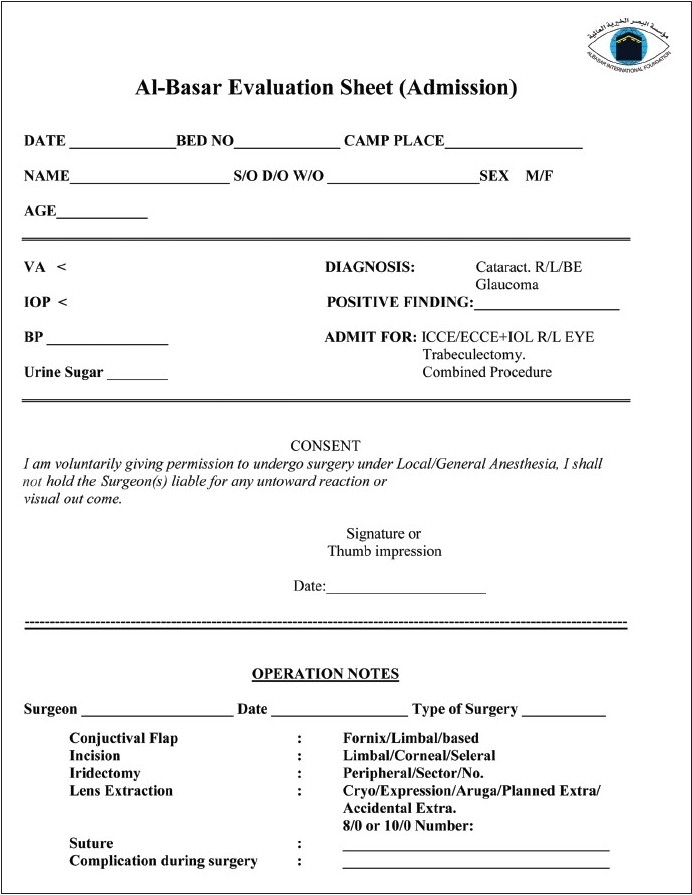
Al-Basar evaluation sheet (Admission)

**Figure 2:**
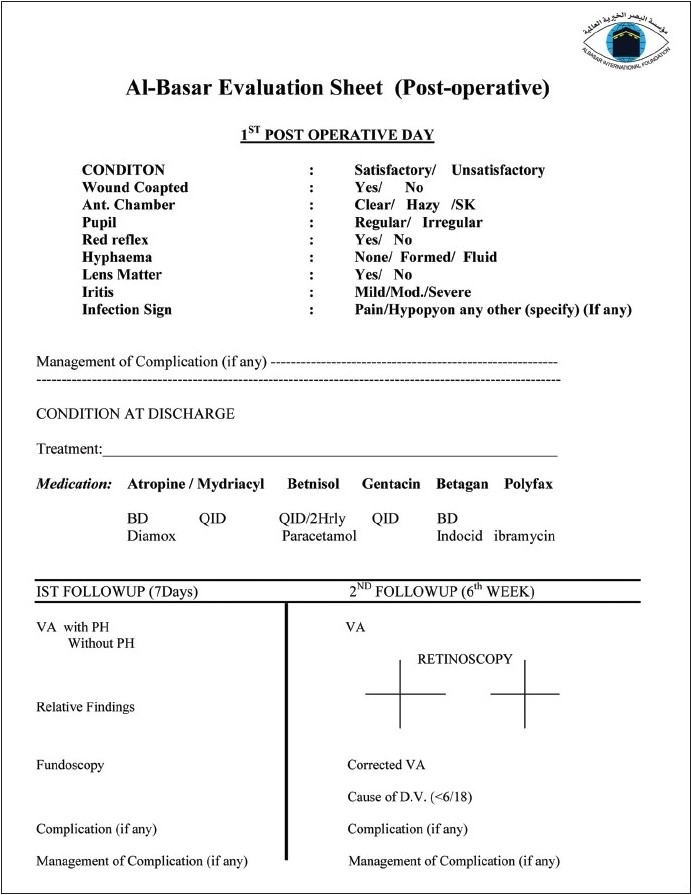
Al-Basar evaluation sheet (Post-operative)

Al-Basar evaluation sheets [Figures 1 and 2] are a concise check list demonstrating the preoperative, intraoperative, and the postoperative data. The consent as well as the medications and the follow-up schedules are also highlighted in this form. In the early 1990s, cataract surgery was performed without IOL implantation. Subsequently, extracapsular cataract extraction with IOL implantation was adopted. Recently, BIF started to implement the most recent technique of phacoemulsification with IOL implantation in the eye camps.

Surgery usually takes 12-15 min, and four to five operating tables are used concurrently. Strict sterilization and disinfection protocols are maintained based on the working conditions at each site. Initial dressing is performed the following day. Examination includes a check list [Figure 2]. Upon discharge, patients receive their postoperative medications, sunglasses, and a simplified instruction pamphlet written in their native language. One BIF doctor and one technician perform the two post-op follow-up visits. The first follow-up is at 1 week postoperatively and the second is 6-8 weeks later. Glasses and necessary medications are dispensed at these visits.

Standard data sheets and a questionnaire specifically designed to compile the patient information were used for data collection. Procedures used to control the quality of data included the training of statistical clerks and the verification of data entry to ensure correct input. These were the only available methods for improving the quality of data as the camp conditions do not allow for “test-retest” or other methods for ensuring the reliability and validity of data. Data were entered into a computer and with the help of biostatistician, the tables were constructed by collapsing response categories for particular variables into broader categories. Descriptive statistics were used to analyze the data. Analysis of the outcomes by country, camp, surgeon, co-morbidity, etc are beyond the scope of this paper.

Study limitations included the large numbers of patient visits and patients screened in a poor security setup, the time factor, tight schedules of campaigns, and the difficult working environment. However, these were addressed as challenges rather than limitations.

Ethical considerations were observed such as obtaining an informed consent from the patient and/or a relative before surgery. All physicians working with BIF were accredited and approved by the local health authorities. The necessary approval to hold the campaign was obtained from the authorities concerned. The work was coordinated with the National Plans and the local “Committee for the Prevention of Blindness.” Services provided during the eye campaigns were ophthalmic surgery, distribution of necessary medicine, glasses and the provision of preventive measures and education.

## RESULTS

Between the periods of November 1989 and June 2006, BIF conducted 620 eye camps. Table 1 presents the distribution of the eye camps by a country. Patients attending the camps, number of surgeries performed, and number of IOLs implanted in each country are also demonstrated. Table 2 summarizes the outcomes of these 16 years of BIF campaigns in 38 countries in Africa, Asia, and the Middle East. A total of 2,155,562 individuals have been examined and/or treated, 186,765 cataract extraction surgeries performed, 96,239 of which had IOL implantation. Spectacles were prescribed for 140,426 individuals to treat aphakia and ametropia in patients who received IOLs implantation. All procedures were “camp” cases. However these camps were held utilizing local available settings, these include tents, school buildings, and other fixed facilities. These were rehabilitated to meet the needs as well the specifications of hygiene and operative room quality. Since 2000, extracapsular cataract extraction with IOL implantation or phacoemulsification with IOL implantation has become the treatment of choice in managing cataract blindness in BIF camps. In all camps, the focus is on cataract because it is the main cause of blindness in these countries. However, other causes of blindness such as entropion, advanced pterygium, glaucoma mandating filtering procedures are managed within the camp premises while corneal scars, retinal pathology, and pediatric ocular muscle misalignment are referred. The numbers for these entities were not available.

**Table 1:** Eye camps held by Al-Basar International Foundation: Country statistics (November 1989–June 2006)

Number	Country	No. of camps	Patients examined	Surgery	Intraocular lense
1	Afghanistan	4	13730	615	474
2	Burkina Faso	7	32770	2603	1052
3	Bangladesh	19	116635	6703	4189
4	Benin	5	29500	2044	1810
5	Cambodia	4	14959	1023	361
6	Cameroon	6	26500	1656	634
7	Central Africa	3	16800	1130	1051
8	Chad	15	85238	5647	4019
9	Djibouti	10	58710	4565	1259
10	Eritrea	3	10490	912	327
11	Ethiopia	7	34300	1969	1778
12	Gambia	4	12078	874	110
13	Ghana	7	32500	1936	1194
14	Guinea	4	19000	1349	954
15	India	27	40746	4688	1469
16	Kashmir	2	5750	333	180
17	Kenya	4	13988	545	278
18	Kurdistan	2	6572	604	58
19	Malawi	2	6900	128	122
20	Mali	9	42000	3668	2250
21	Maldives	1	723	25	18
22	Mauritania	7	27300	1853	846
23	nepal	1	2150	234	0
24	niger	17	72037	6744	2549
25	Nigeria	23	127297	9689	5391
26	Pakistan	255	633500	68481	33965
27	Philippines	2	5694	205	23
28	Rwanda	1	5500	58	54
29	Sierraleone	1	2200	187	15
30	Senegal	4	16700	1530	1199
31	Somalia	15	63129	7328	2333
32	Sri Lanka	6	20500	3492	3443
33	Sudan	100	376636	31940	16271
34	Tanzania	6	15891	1051	165
35	Thailand	1	3621	89	35
36	Togo	7	33000	2181	1422
37	Uganda	2	10750	352	248
38	Yemen	27	119768	8334	4693
Total		620	2,155,562	186,765	96,239

**Table 2:** Summary of the eye camp outcomes in 38 countries

Outcome	Numbers
Countries that benefited	38
Campaigns held	620
Patients examined/treated	2,155,562
Surgeries performed	186,765
Intraocular lenses implanted	96,239
Spectacles distributed (Aphakic + Refractive for IOLs patients)	140426

As shown in Table 3, most of the activities took place in Asia and the Middle East. This makes up 74% of the eye camps, 65% of the patients, and 71% of the surgeries. Alternately, this table also shows that one third of the work performed was in the 23 African countries that were covered.

**Table 3:** Eye camp outcomes by region

Region	No. of countries	No. of camps	Outpatients seen	Surgeries done	Intraocular lense implanted	Glasses given out (aphakic + refractive for IOLs patients)
West Africa	15 (39)	124 (20)	592,220 (27)	44,830 (24)	25,331 (26)	39,320 (28)
Rest of Africa	8 (21)	35 (5.7)	156,529 (7)	9510 (5)	4231 (4)	12,637 (9)
Asia	11 (29)	318 (51)	846,399 (39)	83,788 (45)	43,262 (45)	46,341 (33)
Middle East	4 (11)	143 (23)	560414 (26)	48547 (26)	23,415 (24)	42,128 (30)
Total	38	620	2,155,562	186,765	96,239	140,426

Figures in parentheses are in percentage

In the eye camps, 90-95% of patients presented with bilateral advanced (mature or hyper mature) cataracts. The presenting visual acuity in these patients was usually light perception (LP) or hand motion (HM). Table 4 presents the level of vision restoration in patients after cataract surgery. The majority of the operative patients [110,191 (59%)] regained good vision of 6/6 to 6/18. More than one third [65,368 (35%)] had their vision restored to a borderline level (6/24 to 6/60) and the outcome was poor (< 6/60) in 11,206 (6%).

**Table 4:** Postoperative visual acuity of patients who underwent surgery at Al-Basar International Foundation eye camps

Level of eye sight achieved (BCVA)	No. of patients (%)
6/6 – 6/18	110,91 (59)
6/24 – 6/60	65,368 (35)
< 6/60	11,206 (6)

## DISCUSSION

Globally, cataract constitutes almost half of the causes of DV, followed by glaucoma, age-related macular degeneration (AMD), and diseases such as corneal opacities and diabetic retinopathy. Childhood blindness and trachoma each constituted approximately 4-5% of DV.^1^ The outcome of 16 years of campaigns in 38 countries, demonstrated by the number of campaigns held, patients treated, surgeries performed, IOLs implanted, and spectacles distributed clearly show the size and resources made available by BIF towards achieving its objective of control and preventing blindness. This is far beyond the work performed and services provided by similar foundations. For example a similar foundation performs just over 1200 cataract surgeries per year.^5^ Another foundation performed approximately 50,441 surgeries and covered ten countries in Asia and Africa.^6^

**Figure 3:**
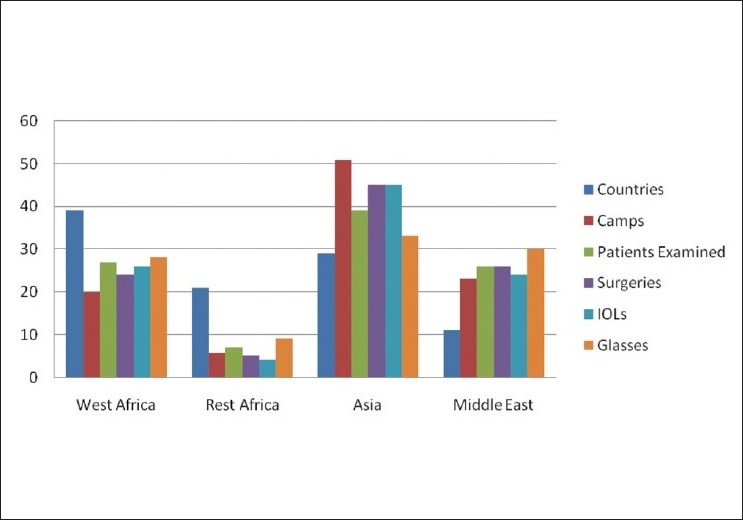
Camps/ camps outcomes by region

**Figure 4:**
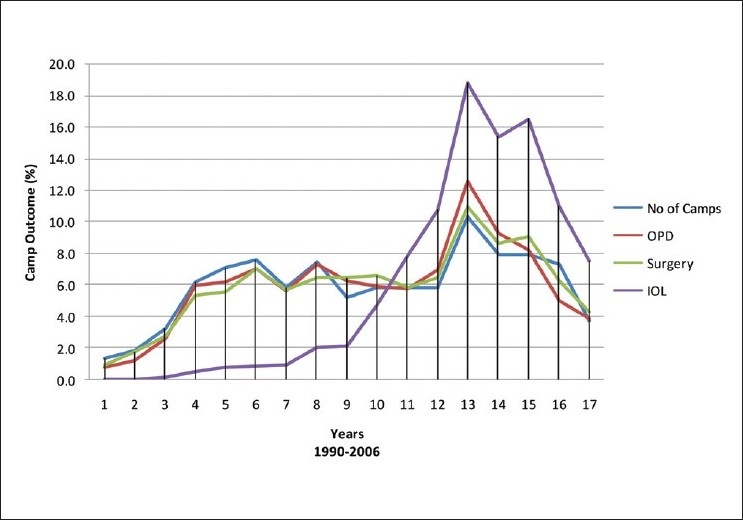
Yearly statistics of the camp outcomes (1990 - 2006)

Although fewer Asian and Middle Eastern (15) compared to African countries (23) were targeted by the BIF, nearly three quarters of the campaigns were held in Asia. This was obviously due to population size and the number of people in need of eye services in Asia. This is also true for the number of patients seen in the out-patient department (OPD), surgeries performed, IOLs implanted, and spectacles dispensed (65%, 71%, 69% and 63%, respectively). The facts that BIF started its work in Pakistan and the Human Resource Development HRD facilities are well established there, are additional factors explaining why Asia in general and Pakistan in specific have the largest numbers of patients and camps. Figure 3 demonstrates the regional distribution of the activities. Separation by country clearly indicates Pakistan, Sudan, Yemen, Nigeria, Bangladesh, and Niger were foremost [Table 1]. A common factor in these countries is the establishment of AL-Basar Base Hospital (secondary or tertiary). Figure 4 shows that over years, there is a generalized trend of progressive increase in the number of camps, patients assessed, surgeries performed, and IOLs implanted. In 1992, only 3% of surgeries involved IOLs implantation compared to 90% in 2005 [Table 5].

**Table 5:** Eye camps statistics/year

S. no.	Year	No. of camps	OPD	Surgery	Intraocular lense
1	1990	8	16289	1703	0
2	1991	11	25313	3277	0
3	1992	20	55219	5077	169
4	1993	38	128614	9926	448
5	1994	44	132945	10350	727
6	1995	47	151458	13117	823
7	1996	36	120591	10640	905
8	1997	46	156146	12118	1972
9	1998	32	133681	12020	2056
10	1999	36	126310	12281	4515
11	2000	36	124194	10902	7583
12	2001	36	148921	12112	10372
13	2002	64	270197	20472	18127
14	2003	49	199333	16100	14818
15	2004	49	176697	16925	15921
16	2005	45	107560	11693	10584
17	2006	23	82094	8052	7219
Total	16	620	2155562	186765	96239

The number of people with DV in Asia is enormous, but resources and facilities in Asia fall far short of the need.^1^ Al-Basar Foundation conducted its first eye campaign in Zanzibar, Africa, in 1991. This likely makes BIF the first eye organization to conduct surgery camps for large groups of patients in Africa.

BIF established its first hospital called Al-Ibrahim Eye Hospital in 1990 in Karachi, Pakistan. All the staff for the camps and other hospitals of the foundation are drawn from this hospital. In 1996, another tertiary base hospital called Maghrebi Eye Complex was established in Khartoum-Sudan as a teaching and training centre. This hospital also provides support and man-power to other centers.

Table 6 illustrates the WHO standards for cataract surgery outcomes.^7^ Work that meets these standards is considered acceptable. Applying these standards to the surgical outcomes of BIF eye camps, the achievement of a good vision in 59% of surgeries, borderline in 35%, and poor in 6% is an indication of the quality of eye care provided in the BIF campaigns. Table 7 demonstrates the underlining causes of poor outcomes in the third group. Intra and post-operative complications constitute a small portion of this number (1321 [11.8%]) compared with other causes. Glaucoma and retinal pathology especially diabetic retinopathy are problems that need special attention. The limited outcome together with the need of long-term follow-up, make them real challenging tasks for eye care providers in developing countries.

**Table 6:** WHO standards for cataract surgery outcomes

Level of eye sight achieved	Proportion of patients (%)
Good (6/6 – 6/18)	55-60
Borderline (6/24 – 6/60)	30-35
Poor (< 6/60)	< 10

**Table 7:** Causes of poor postoperative outcomes of eyes that underwent surgery at Al-Basar International Foundation eye camps

Causes of poor outcome	Number of patients (%)
Intra and post-op. complications	1321 (11.8)
Advanced glaucoma	3740 (33.4)
Retinal pathology	3193 (28.5)
Others	2952 (26.3)
Total	11,206 (100)

Two factors were crucial in achieving these results. The two meticulous post-operative follow-up visits. The first one was within one week and the second was done 6-8 weeks later. The compliance for both visits was excellent; 90-95% and 60-70% respectively. The other factor was the maintenance of strict sterilization and disinfection protocols at all surgical sites which did minimize infection rate.

However, the outcomes may also have been influenced by a variety of other factors such as proper screening techniques,^8^ thorough preoperative evaluation,^6^ strict intraoperative environment, meticulous postoperative follow-up schedule, and the fact that surgeries were performed by MES-oriented cataract surgeons. Notably, BIF conducts its own camps rather than using an intermediary. This has the added advantage of maintaining quality control over the camps and services dispensed throughout Asia and Africa.

In addition to the restoration of vision for more than 2 million people in Africa, Asia, and the Middle East, other outcomes of BIF include the establishment of 21 eye hospitals, bringing up infrastructure, training local workers, motivation, and encouragement of government and non-government sectors as well as the creating public awareness of factors leading to blindness.

In conclusion, the eye campaigns held by BIF as well as other organizations^9^ have helped many developing countries and underprivileged people. The momentum to organize more campaigns and establish hospitals should be maintained. The concept of MES with its emphasis on the prevention of blindness is an outstanding support for the work of the WHO and IAPB. The people of these countries are served irrespective of race, sex, and religion.

It can be concluded from this study that most of DV in BIF camps were fairly easy to manage. The campaigns resulted in the restoration of vision for more than 2 million people. Services provided were of high quality. Despite the fact that the field settings were not ideal, the outcomes matched the IAPB/WHO standards. Results of cataract surgeries in some of the developing countries fall behind these standards.
